# Obesity Inhibits Angiogenesis Through TWIST1-SLIT2 Signaling

**DOI:** 10.3389/fcell.2021.693410

**Published:** 2021-09-29

**Authors:** Tendai Hunyenyiwa, Kathryn Hendee, Kienna Matus, Priscilla Kyi, Tadanori Mammoto, Akiko Mammoto

**Affiliations:** ^1^Department of Pediatrics, Medical College of Wisconsin, Milwaukee, WI, United States; ^2^Department of Cell Biology, Neurobiology and Anatomy, Medical College of Wisconsin, Milwaukee, WI, United States; ^3^Department of Pharmacology and Toxicology, Medical College of Wisconsin, Milwaukee, WI, United States

**Keywords:** angiogenesis, obesity, adipose tissue, TWIST1, SLIT2

## Abstract

Angiogenesis is required for functional adipose tissue maintenance, remodeling, and expansion. Physiologically balanced adipogenesis and angiogenesis are inhibited in subcutaneous adipose tissue in obese humans. However, the mechanism by which angiogenesis is inhibited in obese adipose tissue is not fully understood. Transcription factor TWIST1 controls angiogenesis and vascular function. TWIST1 expression is lower in obese human adipose tissues. Here, we have demonstrated that angiogenesis is inhibited in endothelial cells (ECs) isolated from adipose tissues of obese humans through TWIST1-SLIT2 signaling. The levels of TWIST1 and SLIT2 are lower in ECs isolated from obese human adipose tissues compared to those from lean tissues. Knockdown of TWIST1 in lean human adipose ECs decreases, while overexpression of TWIST1 in obese adipose ECs restores SLIT2 expression. DNA synthesis and cell migration are inhibited in obese adipose ECs and the effects are restored by TWIST1 overexpression. Obese adipose ECs also inhibit blood vessel formation in the gel subcutaneously implanted in mice, while these effects are restored when gels are mixed with SLIT2 or supplemented with ECs overexpressing TWIST1. These findings suggest that obesity impairs adipose tissue angiogenesis through TWIST1-SLIT2 signaling.

## Introduction

Obesity is regarded as a world epidemic with more than 1 billion people overweight in the world (Huang et al., 2016; GBD 2015 Mortality and Causes of Death Collaborators, 2016) and approximately two-thirds of US adults are overweight or obese (Cao, 2010). Obesity is highly associated with increased morbidity and mortality through its association with cardiovascular disease, type 2 diabetes, hypertension, stroke and certain types of cancer (e.g., colorectal, breast, gastrointestinal, and prostate cancer) (Shibata et al., 2004; Despres, 2006; Despres and Lemieux, 2006; Cao, 2010; O’Sullivan et al., 2018).

Subcutaneous adipose tissue is the largest and safest site to store lipid in the body. In a healthy condition, functional adipogenesis, adipocyte differentiation and hyperplastic expansion maintain metabolism in the body. However, in an obese condition, hypertrophic adipose tissue is inflamed and the capacity of lipid storage in the subcutaneous adipose tissue is exceeded, which leads to the accumulation of excess amounts of dysfunctional lipids in the ectopic sites (e.g., visceral fat, peri or epicardial fat, liver, skeletal muscles). The deregulated adipose tissue remodeling in an obese condition results in local and systemic insulin resistance and inflammation, consequently increasing the risk of obesity-related diseases (Crewe et al., 2017; Hammarstedt et al., 2018; Chouchani and Kajimura, 2019).

Well-organized functional angiogenesis is necessary for organ development, regeneration and repair from injury (Mammoto and Mammoto, 2019). Under obese conditions, due to its proinflammatory state, endothelial signaling is dysregulated and EC functionality is impaired (Chudek and Wiecek, 2006; Hammarstedt et al., 2018; O’Sullivan et al., 2018). Obese conditions also promote cellular senescence and cell cycle arrest (Yokoyama et al., 2014; Briot et al., 2018; Gustafson et al., 2019; Conley et al., 2020; Smith et al., 2021). While healthy subcutaneous adipose tissue is maintained by physiologically balanced adipogenesis and angiogenesis, inflammatory and anti-angiogenic signals in hypertrophic obese adipose tissues inhibit angiogenesis and disrupt physiological vascular function, which results in local hypoxia, metabolic stress, and tissue dysfunction (Hosogai et al., 2007; Halberg et al., 2009; Pasarica et al., 2009; Cao, 2010; Christiaens and Lijnen, 2010; Xiao et al., 2016; Crewe et al., 2017; Hammarstedt et al., 2018; Chouchani and Kajimura, 2019). These EC dysfunctions, chronic inflammation and impairment of angiogenesis in obese adipose tissues lead to ectopic lipid accumulation and obesity-associated cardiovascular diseases (Friedman, 2000; Shibata et al., 2004; Hosogai et al., 2007; Pasarica et al., 2009; Cao, 2010; Christiaens and Lijnen, 2010; Crewe et al., 2017; Hammarstedt et al., 2018; Chouchani and Kajimura, 2019). Thus, we need to understand the mechanism by which obesity inhibits angiogenesis in adipose tissues.

TWIST1 is a basic helix-loop-helix transcription factor, which regulates vascular development (Li et al., 2014) and function (Mammoto et al., 2013). TWIST1 is involved in obesity- and angiogenesis-associated diseases such as diabetes (Pettersson et al., 2010), chronic obstructive pulmonary disease (COPD) (Nishioka et al., 2015), cancer (Wang et al., 2011), pulmonary fibrosis (Mammoto et al., 2016), and atherosclerosis (Mahmoud et al., 2016, 2017). TWIST1 also controls cellular metabolism; knockdown of TWIST1 in fat cells stimulates oxygen consumption and mitochondrial biogenesis, which results in resistance to obesity (Pan et al., 2009). Inhibition of TWIST1 activity increases the expression of PGC1α that stimulates mitochondrial biogenesis (Finck and Kelly, 2006; Austin and St-Pierre, 2012; Patten and Arany, 2012; Fan and Evans, 2015) and angiogenesis (Arany et al., 2008; Patten and Arany, 2012; Kluge et al., 2013; Rowe et al., 2014) in fat cells (Pan et al., 2009) and aged ECs (Hendee et al., 2021). The SLIT family proteins are extracellular matrix (ECM) proteins and consist of three members (SLIT1-3). SLIT proteins are known as axon guidance molecules and control brain development (Nguyen Ba-Charvet et al., 1999; Wang et al., 1999; Brose and Tessier-Lavigne, 2000). Recently it is reported that SLIT2 promotes thermogenic activity in beige fat tissue and the levels of SLIT2 decrease in the fat tissue of obese mice (Svensson et al., 2016). SLIT2 is expressed in various types of cells including ECs (Urbich et al., 2009; Guijarro-Munoz et al., 2012; Rama et al., 2015; Tavora et al., 2020) and is involved in physiological and pathological angiogenesis (Rama et al., 2015; Liu et al., 2018; Genet et al., 2019; Tavora et al., 2020). However, the role of endothelial TWIST1 and SLIT2 in obese adipose tissue angiogenesis remains unclear.

Here we found that angiogenesis is impaired in adipose tissue ECs isolated from obese humans through TWIST1-SLIT2 signaling. The levels of TWIST1 and SLIT2 are lower in obese adipose ECs and overexpression of TWIST1 or treatment with SLIT2 stimulates EC DNA synthesis and migration *in vitro* and vascular formation in the gel implanted on mice. Modulating TWIST1-SLIT2 signaling in ECs could be a novel therapeutic approach for obesity and obesity-associated diseases.

## Materials and Methods

### Materials

Recombinant human SLIT2 (aa1122–1529) was purchased from R&D Systems (Minneapolis, MN, United States). Anti-β-actin (A5316) monoclonal antibody was from Sigma (St. Louis, MO, United States). Anti-VEGFR2 (2479) antibody was from Cell Signaling (Danvers, MA, United States). Anti-TWIST1 antibody was from Abcam (ab50887, Cambridge, MA, United States) and Santa Cruz Biotechnology (sc-15393, Dallas, TX, United States).

### Adipose Endothelial Cell Isolation and Culture

Human subcutaneous adipose tissues [*n* = 29, body mass index (BMI) < 30 or >30] were obtained as discarded surgical specimens from patients undergoing abdominal surgeries. After surgical removal, samples were placed in ice-cold HEPES buffer and immediately transferred to the laboratory for EC isolation. De-identified patient demographic data were collected using the Generic Clinical Research Database (GCRD) at the Medical College of Wisconsin (MCW). All protocols were approved by the Institutional Review Board of MCW and Froedtert Hospital. The information about sex, age, and BMI of each sample is listed in Table 1 and sample demographic information is summarized in Table 2. Human adipose ECs were isolated as previously described and cultured in 10% FBS/ECM (Mammoto et al., 2018, 2019c, 2020; Hendee et al., 2021). All cell culture experiments were conducted between passages 2–3.

**TABLE 1:** Sample information.

**BMI < 30**	**Sex**	**Age**	**BMI**
1	F	47	28
2	M	33	28
3	F	42	26
4	F	47	27
5	F	46	24
6	F	42	27
7	F	51	24
8	F	41	23
9	F	38	29
10	F	30	25
11	F	34	23
12	F	41	24
13	F	33	24
14	F	55	21
15	F	31	19

**BMI > 30**	**Sex**	**Age**	**BMI**

1	F	50	34
2	F	46	36
3	F	44	37
4	F	32	34
5	F	38	37
6	F	50	44
7	F	36	37
8	F	43	35
9	F	37	31
10	M	40	32
11	F	43	34
12	F	30	45
13	F	43	41
14	F	45	37

**TABLE 2:** Sample demographics.

**Sample demographics (*n* = 29)**	**Lean (BMI < 30, *n* = 15)**	**Obese (BMI > 30, *n* = 14)**
Gender, Male/Female	1 (6.6%)/14 (93.4%)	(7.1%)/13 (92.9%)
Age, year (mean ± SEM)	40.73 ± 1.95	41.21 ± 1.61
Body mass index (mean ± SEM)	24.78 ± 0.71	36.81 ± 1.11
Underlying diseases		
Coronary artery disease	0 (0%)	1 (7.1%)
Hypertension	0 (0%)	5 (35.7%)
Hyperlipidemia	0 (0%)	2 (14.2%)
Cancer	1 (6.6%)	3 (21.4%)

### Molecular Biology and Biochemistry Experiments

RNA isolation was performed using an RNeasy mini kit (Qiagen, Valencia, CA, United States). Quantitative reverse transcription (qRT)-PCR was performed using the iScript reverse transcription and iTaq SYBR Green qPCR kit (Bio-Rad, Hercules, CA, United States) then analyzed using the real-time PCR system (Bio-Rad). β2 microglobulin (B2M) was used for overall cDNA content. The primers used for human B2M and TWIST1 are described before (Mammoto et al., 2018, 2020; Hendee et al., 2021). Primers for human SLIT2 are forward; AGCCGAGGTTCAAAAACGAGA, reverse; GGCAGTGCAAAACACTACAAGA. The protein levels of human SLIT2 were measured using ELISA (MyBioSource, San Diego, CA, United States).

### Gene Manipulation

Gene knockdown was conducted by siRNA transfection using siLentFect (Bio-Rad) or lentiviral transduction. Human TWIST1 siRNA was described previously (Mammoto et al., 2020; Hendee et al., 2021). siRNA with an irrelevant sequence (QIAGEN) was used as a control. Lentiviral construct targeting human SLIT2 (SLIT2 shRNA) were CCGGCCTGGAGCTTTCTCACCATATCTCGAGATATGGTG AGAAAGCTCCAGGTTTTTG (MilliporeSigma). Generation of lentiviral vectors was accomplished by a five-plasmid transfection procedure as reported (Mammoto et al., 2009, 2018, 2019b, 2020). Human adipose ECs were incubated with viral stocks in the presence of 5 μg/ml polybrene (Sigma) and 90–100% infection was achieved 3 days later (Mammoto et al., 2009, 2018, 2019b, 2020). Lentivirus with vector alone was used as a control.

### Cell Biological Analysis

Human adipose ECs were seeded at a density of 1 × 10^5^ cells/35 mm dish and DNA synthesis was measured using the Click-iT^TM^ EdU Cell Proliferation Kit (Thermofisher, Waltham, MA, United States) (Hendee et al., 2021). The total cells were labeled with Hoechst nuclear dye and cells were imaged using a Nikon A1R confocal laser scanning microscope. Quantification was performed using ImageJ software (NIH). EC migration was analyzed by seeding human adipose ECs (1 × 10^5^ cells/100 μl) in a trans-well chamber (Corning Costar) coated with 0.5% gelatin. 5% FBS was added to the lower chamber to promote migration and cells were incubated for 16 h (Hendee et al., 2021). Cells migrated to the opposite side of the membrane were stained with Wright Giemsa solution (Fisher Scientific) and quantified. The number of surviving cells were counted using trypan blue staining, in which ECs were treated with 0.1% trypan blue (Sigma) in PBS and counted under a light microscope (Salerno et al., 2011; Chen et al., 2015).

### Mouse Subcutaneous Gel Implantation

The *in vivo* animal study was carried out in accordance with the recommendations in the Guide for the Care and Use of Laboratory Animals of the National Institutes of Health. The protocol was reviewed and approved by the Animal Care and Use Committee of MCW. NOD SCID gamma (NSG) mice (Jackson Laboratory, stock#, 5557) were used for the study. Human adipose ECs were treated with viral stock expressing GFP for labeling and the transduction efficiency was confirmed before the assay. Fibrin gel was fabricated as previously described, and GFP-labeled human adipose ECs (1 × 10^6^ cells), in which gene expression was manipulated, and human fibroblasts (3 × 10^5^ cells) were mixed in the gel (Mammoto et al., 2018, 2019b, 2020; Hendee et al., 2021). The drops of gels were incubated at 37°C for 30 min and subcutaneously implanted onto the back of the NSG mice (Supplementary Figure 2A). The gel was harvested 7 days after implantation, fixed with 4% paraformaldehyde, cryosectioned, and immunohistochemical (IHC) analysis was conducted as described previously (Mammoto et al., 2018, 2019b, 2020; Hendee et al., 2021). The IHC images were taken using a Nikon A1R confocal laser scanning microscope and stacks of optical sections (20-μm thick) were compiled to form three-dimensional images using software associated with a confocal microscope. Morphometric analysis was performed using ImageJ and AngioTool software. Vascular network formation of GFP-labeled human adipose ECs was evaluated by measuring the area of GFP-labeled blood vessels from five different areas of the gel.

### Microarray Data Analysis

Publicly available datasets from eight obese and seven lean adult human subcutaneous adipose samples (NCBI GEO, GSE55200) were utilized, and differential gene expression analysis and volcano plot generation were performed by GEO2R. The total number of genes identified by the array and displayed on the volcano plot was 48,242, with 26,391 being downregulated and 21,851 upregulated. Of these, 859 downregulated and 1,151 upregulated genes possessed adjusted *p*-values < 0.05 following Benjamini and Hochberg false discovery rate multiple-testing correction of *p*-values, resulting in a total of 2,010 significantly differentially expressed genes. Thousand three hundred and seventy four of these genes were assigned a GenBank Accession ID. Upon ID conversion to official gene symbol using the Database for Annotation, Visualization, and Integrated Discovery (DAVID) v6.8, 1,302 genes returned with unique conversion IDs. A subset of genes had multiple transcripts appear on the array sharing the same accession ID; these transcripts all changed in the same direction by gene and were encompassed by including the converted gene names only once in the final gene count. Thus, 548 downregulated and 754 upregulated significantly expressed genes underwent Biological Processes Gene Ontology (BP GO) Term analysis through the Functional Annotation Chart tool of the DAVID software. All down and upregulated genes identified in the top 30 BP GO Term categories by *p*-value were made into a network and color-coded using Ingenuity Pathway Analysis (IPA) software. The network mapped the shortest interactions among TWIST1 and the genes from the Top 30 BP GO Term categories, SLIT2 and the Top 30 BP GO Term genes, and the TWIST1 and SLIT2 groups and the Top 30 BP GO Term genes.

### Statistics

All phenotypic analysis was performed by masked observers unaware of the identity of experimental groups. Power analysis was conducted to provide 80% power to detect an effective 20–30% difference between the experimental groups. Error bars (SEM) and *p*-values were determined from the results of three or more independent experiments. Student’s *t*-test was used for statistical significance for two groups. For more than two groups, one-way ANOVA with a *post hoc* analysis using the Bonferroni test was conducted.

## Results

### TWIST1 and SLIT2 Expression Is Inhibited in Obese Adipose Endothelial Cells

**FIGURE 1:**
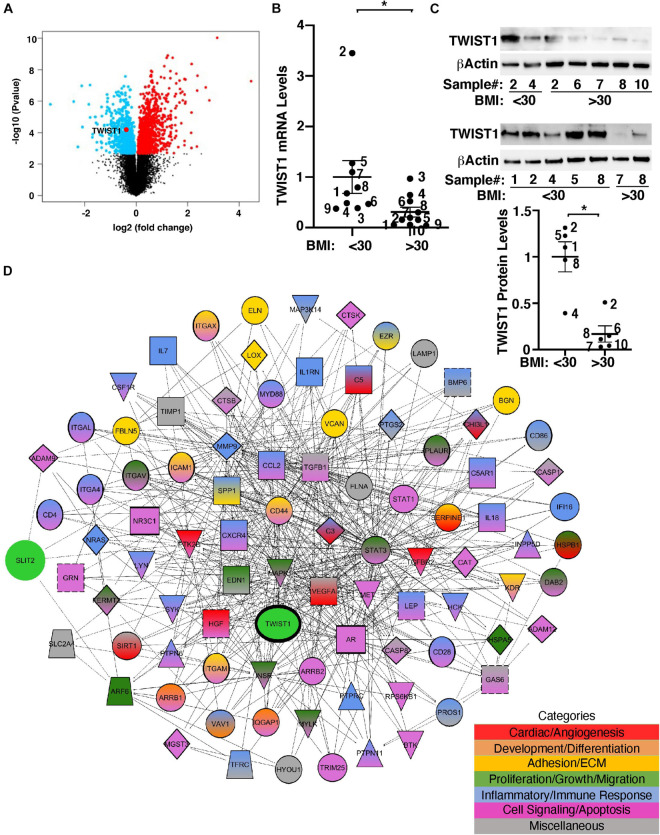
TWIST1 expression in obese human subcutaneous adipose ECs is lower than lean ECs. **(A)** Volcano plot of differentially expressed genes, including TWIST1, in eight obese vs. seven lean adult human subcutaneous adipose samples from the GSE55200 dataset. Blue indicates significantly down-regulated genes and red indicates significantly up-regulated genes with Benjamini and Hochberg adjusted *p*-values < 0.05. **(B)** Graph showing the TWIST1 mRNA levels in ECs isolated from lean (BMI < 30) vs. obese (BMI > 30) human adipose tissues (*n* = 9–10, mean ± SEM, *p < 0.05). The numbers along the dots correspond to the clone numbers in Table 1. **(C)** Immunoblots showing the TWIST1 and β-Actin protein levels in ECs isolated from lean (BMI < 30) vs. obese (BMI > 30) human adipose tissues (*n* = 5, mean ± SEM, *p < 0.05). The numbers along the dots correspond to the clone numbers in Table 1. **(D)** Network showing connections among TWIST1, SLIT2, and genes from the top 30 BP GO Term categories derived from 1,302 significantly differentially expressed down and upregulated genes with Benjamini and Hochberg adjusted *p*-values < 0.05 from the GSE55200 dataset. Genes are color-coded by GO Term categories. Red, angiogenesis-related genes; Orange, development-related genes; Gold, ECM and cell adhesion-related genes; Dark green, cell proliferation, migration-related genes; Blue, inflammation and/or immune response-related genes; Purple, cell signaling and/or apoptosis-related genes; Gray, miscellaneous category-related genes; Black outlines, genes from the GSE55200 dataset.

TWIST1 controls cellular metabolism and is involved in obesity- and angiogenesis-associated diseases (Pettersson et al., 2010; Wang et al., 2011; Nishioka et al., 2015; Mahmoud et al., 2016; Mammoto et al., 2016; Mahmoud et al., 2017). It has been reported that TWIST1 mRNA expression is lower in the adipose tissues from obese humans (Pettersson et al., 2011). Consistently, the levels of TWIST1 were significantly lower in obese human adipose tissues when analyzed using the unbiased publicly available microarray dataset (GSE55200) (Figure 1A). To specifically examine the effects of obesity on TWIST1 expression in ECs, we isolated ECs from obese (BMI > 30) vs. lean (BMI < 30) human subcutaneous adipose tissues and measured the mRNA levels of TWIST1. The levels of TWIST1 mRNA in ECs isolated from obese human adipose tissues (BMI > 30) were 71% lower than those in lean human adipose ECs (Figure 1B). We confirmed the results using immunoblotting (IB) showing that the TWIST1 protein levels were also 83% lower in ECs isolated from obese human adipose tissues (BMI > 30) compared to those in lean human adipose ECs (Figure 1C).

**FIGURE 2:**
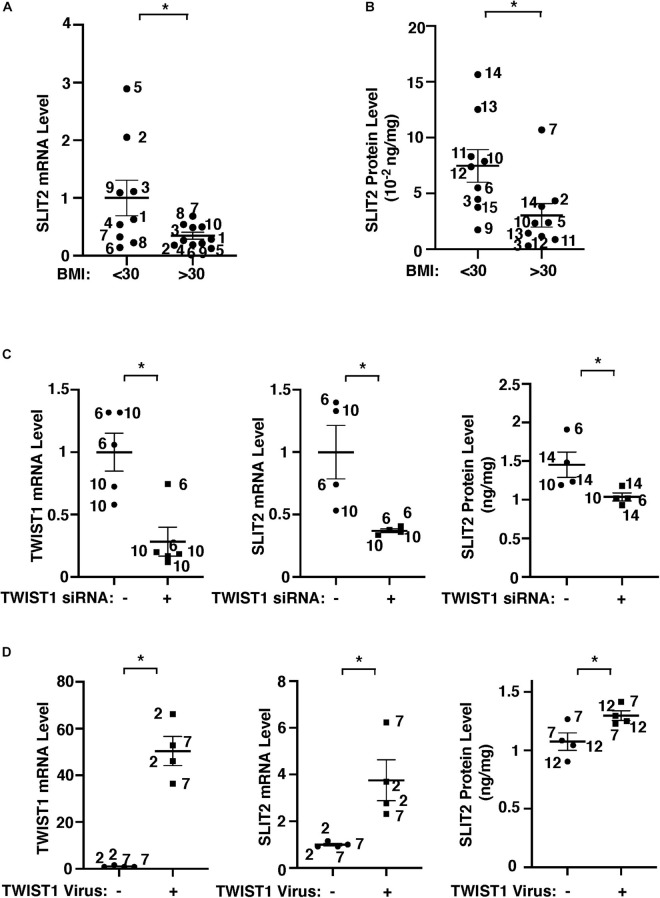
TWIST1 controls SLIT2 expression in lean vs. obese adipose ECs. **(A)** Graph showing SLIT2 mRNA expression in lean vs. obese human adipose ECs (*n* = 9–10, mean ± SEM, *p < 0.05). **(B)** Graph showing SLIT2 protein expression in lean vs. obese human adipose tissues (*n* = 9, mean ± SEM, *p < 0.05). **(C)** Graphs showing mRNA expression of TWIST1 and SLIT2 in lean human adipose ECs treated with TWIST1 siRNA or control siRNA with irrelevant sequences (*n* = 4–5, mean ± SEM, *p < 0.05). Graph showing protein expression of SLIT2 in lean human adipose ECs treated with TWIST1 siRNA or control siRNA with irrelevant sequences (*n* = 4, mean ± SEM, *p < 0.05). **(D)** Graphs showing mRNA expression of TWIST1 and SLIT2 in obese human adipose ECs treated with lentivirus overexpressing TWIST1 or control virus (*n* = 4, mean ± SEM, *p < 0.05). Graph showing protein expression of SLIT2 in obese human adipose ECs treated with lentivirus overexpressing TWIST1 or control virus (*n* = 4, mean ± SEM, *p < 0.05). The numbers along the dots correspond to the clone numbers in Table 1.

Gene network analysis of microarray dataset (GSE55200) reveals that genes from the top 30 GO Term categories derived from 1,302 significantly differentially expressed genes of lean vs. obese adipose tissues, including angiogenesis, development/differentiation, cell proliferation, adhesion, and inflammatory molecules directly or indirectly interacted with TWIST1 (Figure 1D). SLIT2 controls angiogenesis (Rama et al., 2015; Liu et al., 2018; Genet et al., 2019; Tavora et al., 2020) and the levels of Slit2 decrease in the fat tissue of obese mice (Svensson et al., 2016). Consistently, significantly differentially expressed genes of lean vs. obese adipose tissues that interacted with TWIST1 also interacted with SLIT2 (Figure 1D). Thus, we next examined the effects of obesity on SLIT2 expression in human adipose ECs. The levels of SLIT2 mRNA in ECs isolated from obese human adipose tissues (BMI > 30) were 66% lower than those in lean human adipose ECs (Figure 2A). We also confirmed the results using ELISA; the SLIT2 protein levels in obese human adipose tissue were 60% lower than those in lean adipose tissue (Figure 2B).

We next examined whether TWIST1 controls SLIT2 expression in lean and obese adipose ECs. siRNA-based knockdown of TWIST1 in human lean adipose ECs, which downregulated TWIST1 expression (Figure 2C and Supplementary Figure 1A), decreased the SLIT2 mRNA and protein expression by 64% and 29%, respectively (Figure 2C), while TWIST1 overexpression using lentiviral transduction upregulated the SLIT2 expression in obese ECs (Figure 2D). These results suggest that downregulation of TWIST1 decreases SLIT2 expression in obese ECs.

### TWIST1 and SLIT2 Control DNA Synthesis and Migration in Obese Endothelial Cells *in vitro*

**FIGURE 3:**
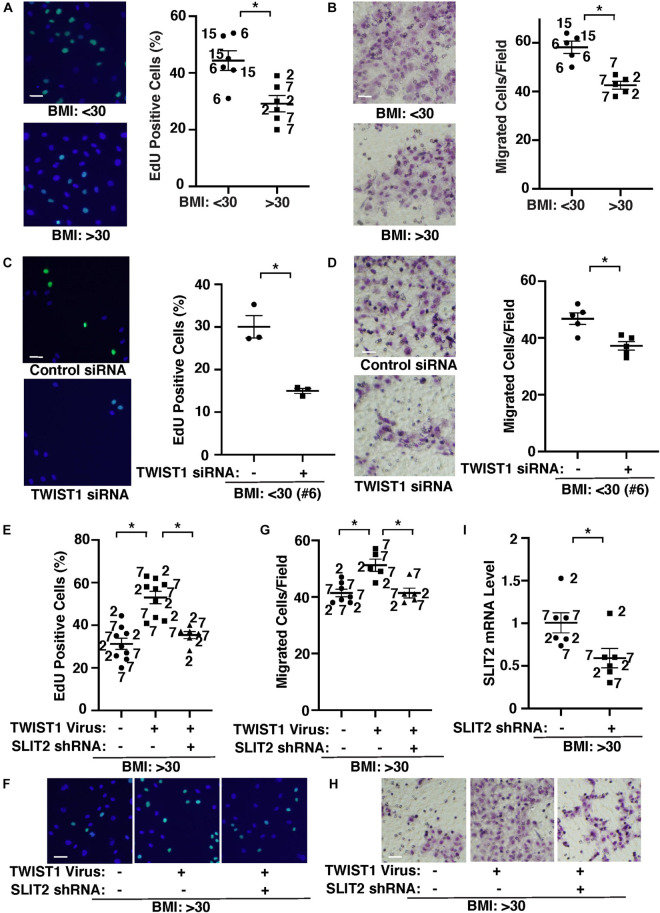
TWIST1-SLIT2 signaling controls DNA synthesis and EC migration in lean vs. obese human adipose ECs. **(A)** Representative images showing EdU positive (green) lean (BMI < 30) vs. obese (BMI > 30) human adipose ECs (*left*). Scale bar, 50 μm. Graph showing DNA synthesis of lean (BMI < 30) vs. obese (BMI > 30) human adipose ECs analyzed using an EdU staining assay (*right*, *n* = 6, mean ± SEM, *p < 0.05). **(B)** Representative images showing lean (BMI < 30) vs. obese (BMI > 30) human adipose ECs migrated to the opposite side of the *trans*-well membrane and stained with Wright Giemsa solution (*left*). Scale bar, 50 μm. Graph showing lean vs. obese human adipose ECs migrating toward 5% FBS (*right*, *n* = 5, mean ± SEM, *p < 0.05). **(C)** Representative images showing EdU positive (green) lean (BMI < 30) human adipose ECs treated with TWIST1 siRNA (*left*). Scale bar, 50 μm. Graph showing EdU-positive lean (BMI < 30) human adipose ECs (clone #6) treated with TWIST1 siRNA (*right*, *n* = 3, mean ± SEM, *p < 0.05). As a control, human adipose ECs were treated with siRNA with irrelevant sequences. **(D)** Representative images showing lean (BMI < 30) human adipose ECs treated with TWIST1 siRNA, migrated to the opposite side of the trans-well membrane and stained with Wright Giemsa solution (*left*). Scale bar, 50 μm. Graph showing lean human adipose ECs (clone #6) treated with TWIST1 siRNA migrating toward 5% FBS (*right*, *n* = 5, mean ± SEM, *p < 0.05). As a control, human adipose ECs were treated with siRNA with irrelevant sequences. **(E)** Graph showing EdU-positive obese (BMI > 30) human adipose ECs treated with lentivirus overexpressing TWIST1 or in combination with SLIT2 shRNA (*n* = 6–9, mean ± SEM, *p < 0.05). As a control, human adipose ECs were treated with control virus (vector alone). **(F)** Representative images showing EdU positive obese (BMI > 30) human adipose ECs treated with lentivirus overexpressing TWIST1 or in combination with SLIT2 shRNA. Scale bar, 50 μm. **(G)** Graph showing obese human adipose ECs treated with lentivirus overexpressing TWIST1 or in combination with SLIT2 shRNA migrating toward 5% FBS (*n* = 5–7, mean ± SEM, *p < 0.05). As a control, human adipose ECs were treated with control virus (vector alone). **(H)** Representative images showing obese (BMI > 30) human adipose ECs treated with lentivirus overexpressing TWIST1 or in combination with SLIT2 shRNA, migrated to the opposite side of the *trans*-well membrane and stained with Wright Giemsa solution. Scale bar, 50 μm. **(I)** Graph showing SLIT2 mRNA levels in obese human adipose ECs treated with lentivirus expressing SLIT2 shRNA (*n* = 6, mean ± SEM, *p < 0.05). As a control, human adipose ECs were treated with control virus (vector alone). The numbers along the dots correspond to the clone numbers in Table 1.

It is reported that capillary density is reduced in obese subcutaneous adipose tissues compared with those in lean animals (Gavin et al., 2005; Halberg et al., 2009; Pasarica et al., 2009; Gealekman et al., 2011; Spencer et al., 2011; Xiao et al., 2016; Crewe et al., 2017; Hammarstedt et al., 2018; Chouchani and Kajimura, 2019). Therefore, we next examined the effects of obesity on EC behaviors and whether TWIST1-SLIT2 signaling mediates EC behaviors in obese adipose ECs. DNA synthesis measured by an EdU nuclear incorporation assay decreased by 37% in obese human adipose ECs (Figure 3A). EC migration analyzed using a transwell migration assay also decreased by 31% in obese human adipose ECs (Figure 3B). When we manipulated the expression of TWIST1 in lean (BMI < 30) vs. obese (BMI > 30) human adipose ECs using siRNA transfection or lentiviral transduction, TWIST1 knockdown, which also suppresses EC survival (Supplementary Figure 1B; Salerno et al., 2011; Chen et al., 2015), inhibited DNA synthesis and EC migration in lean human adipose ECs by 54 and 18%, respectively (Figures 3C,D), while TWIST1 overexpression stimulated DNA synthesis and EC migration in obese human adipose ECs (Figures 3E–H). TWIST1 overexpression-induced stimulation of DNA synthesis and migration in obese human adipose ECs was inhibited when treated with SLIT2 shRNA, which inhibits SLIT2 expression, in combination (Figure 3I), suggesting that endothelial TWIST1 mediates inhibition of DNA synthesis and EC migration in obese adipose ECs through SLIT2.

### TWIST1 and SLIT2 Control Vascular Network Formation in Obese Endothelial Cells *in vivo*

**FIGURE 4:**
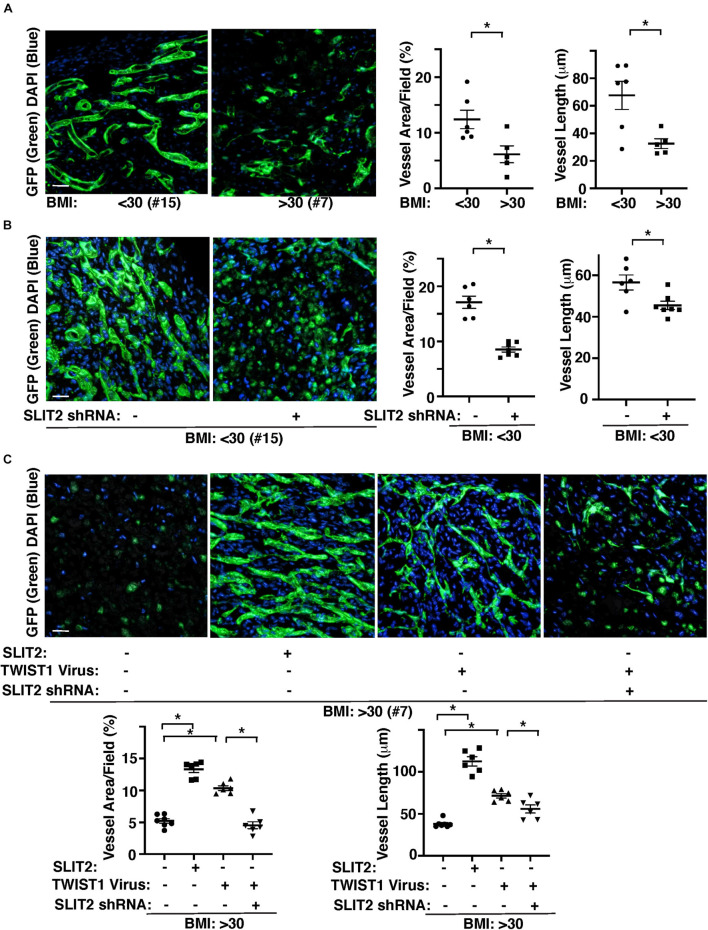
TWIST1-SLIT2 signaling mediates obesity-dependent inhibition of vascular formation in the gel subcutaneously implanted on mice. **(A)** 3D reconstructed immunofluorescence (IF) images showing GFP-labeled vascular formation and DAPI in the fibrin gel supplemented with GFP-labeled lean (BMI < 30, clone #15) vs. obese (BMI > 30, clone #7) human adipose ECs and subcutaneously implanted on NSG mice for 7 days. Scale bar, 50 μm. Graphs showing vascular area and average vessel length in the gel (*n* = 5–6, mean ± SEM, *p < 0.05). **(B)** 3D reconstructed IF micrographs showing GFP-labeled vascular formation and DAPI in the fibrin gel supplemented with GFP-labeled lean human adipose ECs (clone #15) treated with lentivirus encoding SLIT2 shRNA and subcutaneously implanted on NSG mice for 7 days. As a control, lean human adipose ECs were treated with control virus. Scale bar, 50 μm. Graphs showing vascular area and average vessel length in the gel (*n* = 6–7, mean ± SEM, *p < 0.05). **(C)** 3D reconstructed IF micrographs showing GFP-labeled vascular formation and DAPI in the fibrin gel supplemented with GFP-labeled obese human adipose ECs (clone #7) treated with SLIT2 protein, lentivirus overexpressing TWIST1 or in combination with SLIT2 shRNA and subcutaneously implanted on NSG mice for 7 days. As a control, obese human adipose ECs were treated with control virus (vector alone), control shRNA, or control vehicle. Scale bar, 50 μm. Graphs showing vascular area and average vessel length in the gel (*n* = 6–7, mean ± SEM, *p < 0.05).

We also examined the effects of obesity on vascular formation using the mouse gel implantation system, in which fibrin gels supplemented with GFP-labeled lean (BMI < 30) vs. obese (BMI > 30) human adipose ECs were subcutaneously implanted on the back of the immunocompromised NSG mice for 7 days (Supplementary Figure 2A; Mammoto et al., 2019c; Hendee et al., 2021). Consistent with *in vitro* study (Figure 3), vessel formation derived from GFP-labeled supplemented ECs in the cryosectioned gel was attenuated when gel mixed with GFP-labeled obese human adipose ECs was implanted for 7 days; GFP-labeled vascular area and average vessel length were 46 and 55% lower than that in the gel mixed with lean human adipose ECs (Figure 4A). Knockdown of SLIT2 in supplemented lean human adipose ECs suppressed vascular formation in the implanted gel (Figure 4B). The supplemented GFP-labeled VEGFR2^+^ lean human ECs were integrated with host-derived VEGFR2^+^ ECs in the gel, however these effects were attenuated in the gel supplemented with obese ECs or lean ECs treated with SLIT2 shRNA (Supplementary Figure 2B). Supplementation of recombinant SLIT2 protein or overexpression of TWIST1 in supplemented ECs in the gel restored obese EC-derived blood vessel formation and integration with host-derived ECs in the gel, while knockdown of SLIT2 suppressed restoration of vascular formation and integration with host ECs induced by TWIST1 in obese ECs in the implanted gel (Figure 4C and Supplementary Figure 2C). These results indicate that TWIST1 restores obesity-induced disruption of vascular formation through SLIT2.

## Discussion

Impaired angiogenesis and vascular dysfunction in obese adipose tissues contribute to obesity-related diseases. In this report, we have used ECs isolated from lean vs. obese human subcutaneous adipose tissues and demonstrated that vascular formation is inhibited in obese ECs through TWIST1-SLIT2 signaling. The levels of TWIST1 and SLIT2 are lower in obese human adipose ECs compared to those in lean adipose ECs. EC DNA synthesis and migration *in vitro* and blood vessel formation in the gel subcutaneously implanted on the back of mice are suppressed in obese ECs, while TWIST1 overexpression or treatment with SLIT2 protein restores the effects. These results suggest that obesity impairs angiogenesis in subcutaneous adipose ECs through TWIST1-SLIT2 signaling. Modulation of this pathway may be an effective strategy for obesity-related diseases.

While hyperplastic expansion of the adipose tissues maintains healthy metabolism and protects against obesity-related diseases, adipocyte hypertrophy, which is frequently associated with development of obesity (Verboven et al., 2018), leads to adipose tissue dysfunction, inflammation and ectopic lipid accumulation, resulting in the metabolic diseases (Crewe et al., 2017; Hammarstedt et al., 2018; Chouchani and Kajimura, 2019). Impaired angiogenesis is one of the characteristics of hypertrophic adipose tissues. We have found that TWIST1 expression is lower in obese human adipose ECs (Figure 1) and overexpression of TWIST1 restores angiogenic activity and blood vessel formation through SLIT2 in obese ECs (Figures 3, 4). Angiogenesis is controlled by multiple angiogenic factors, which is necessary for well-organized physiological blood vessel formation (Carmeliet and Jain, 2011; Chung and Ferrara, 2011; Herbert and Stainier, 2011). TWIST1 interacts with a number of signaling molecules. For example, TWIST1 mediates age-dependent decline in angiogenesis through VEGFR2 expression (Hendee et al., 2021). Increased levels of TWIST1 contribute to pulmonary fibrosis and lung injury through Tie2 signaling (Mammoto et al., 2013, 2016). TWIST1 also mediates hypoxia-induced pulmonary hypertension by endothelial-to-mesenchymal transition (EndMT) as well as by changing PDGFB expression in ECs (Mammoto et al., 2018, 2020). TWIST1 controls multiple other angiogenic pathways [e.g., VEGF-VEGFR2 (Li et al., 2014), HIF1α (Yang and Wu, 2008), Wnt (Guo et al., 2007), Notch (Chen et al., 2014; Wirrig and Yutzey, 2014), PI3K-AKT (Cheng et al., 2008; Xue et al., 2012), and TGF-β (Mammoto et al., 2018)]. In fact, gene ontology and gene network analysis reveal that TWIST1 interacts with a number of genes that are significantly altered in obese human subcutaneous adipose tissues (Figure 1D). These genes directly or indirectly interact with SLIT signaling molecules as well as other genes to modulate cell signaling, proliferation, ECM structures and control angiogenesis. Thus, TWIST1-SLIT2 signaling would be one of the important targets to control angiogenesis in obese ECs. In addition to angiogenic signaling, TWIST1 also binds to PGC1α, which controls mitochondrial biogenesis and metabolism, and inhibits its co-transcriptional activity in fat cells (Pan et al., 2009). We also reported that TWIST1-PGC1α signaling in ECs contributes to age-dependent disruption of angiogenesis (Hendee et al., 2021). TWIST1 may regulate metabolic and mitochondrial signaling and indirectly control angiogenesis. TWIST1 is also involved in DNA methylation that is associated with obesity (Dahl and Guldberg, 2007; Albuquerque et al., 2015; McCullough et al., 2016). Thus, multiple TWIST1 signaling pathways are involved in angiogenesis in obese conditions.

It is reported that TWIST1 is expressed in different types of adipose tissues (brown, subcutaneous, and visceral) (Pan et al., 2009; Pettersson et al., 2010; Dobrian, 2012; Svensson et al., 2016). SLIT2 is also expressed in all kinds of adipose tissues and the expression is suppressed in subcutaneous adipose tissue from high-fat diet-treated obese mice (Svensson et al., 2016). In addition to TWIST1, the levels of TWIST2 are also lower in obese human subcutaneous adipose tissues in another dataset (GSE15524, not shown). The distribution pattern of TWIST1 and TWIST2 seems to be different; the levels of TWIST1 are higher in subcutaneous adipose tissues compared to visceral adipose tissues, which is strongly correlated with BMI and insulin resistance, while Twist2 is more ubiquitously expressed in the body (Pan et al., 2009; Pettersson et al., 2010). Although it remains unknown how the distribution pattern is regulated, TWIST1 may play important roles in development and remodeling of adipose tissues in an obese condition. While our results demonstrate that overexpression of TWIST1 in obese human adipose ECs restores vascular formation (Figures 3, 4), which may restore adipose tissue homeostasis, it is reported that transgenic mice overexpressing Twist1 in the adipose tissue are susceptible to obesity (Pan et al., 2009). This may be because of the differences in the role of TWIST1 depending on cell types and animal species. TWIST1 is expressed in other cell types such as fibroblasts and epithelial cells (Pozharskaya et al., 2009; Yeo et al., 2018) as well, which also alters the effects of TWIST1 in adipose tissues. Further investigation using endothelial-specific Twist1 knockout/overexpressing mice will elucidate the mechanism.

Crosstalk between angiogenesis and adipogenesis is required for the physiological expansion of the adipose tissue, while these processes are disrupted in obese conditions. Hypertrophic obesity is associated with biological pathways related to hypoxia and inflammation (Gustafson et al., 2009; Crewe et al., 2017; Hammarstedt et al., 2018; Chouchani and Kajimura, 2019), which promotes insulin resistance and lipolysis (Tilg and Moschen, 2006; Guilherme et al., 2008; Galic et al., 2010) and inhibits angiogenesis by secreting proinflammatory molecules (e.g., TNFα, NFkB, JNK) and adipokines (Guilherme et al., 2008; Vazquez-Vela et al., 2008; Crewe et al., 2017; Hammarstedt et al., 2018; Chouchani and Kajimura, 2019). Inhibition of angiogenesis due to inflammatory response may feedback to further develop hypoxia in hypertrophic adipose tissues. It is reported that TWIST1 promotes inflammatory pathways, which leads to various pathological conditions such as atherosclerosis, nephropathy, and pulmonary fibrosis (Tan et al., 2017; Hradilkova et al., 2019; Mahmoud et al., 2019; Ren et al., 2020). Binding of SLIT2 with ROBO receptors also triggers proinflammatory signaling (Zhao et al., 2014; Wang et al., 2020). Thus, TWIST1-SLIT2 signaling may control subcutaneous adipose tissue angiogenesis through inflammatory signaling as well.

Adipogenesis in subcutaneous adipose tissues is also regulated by production of ECM proteins and their mechanics (Hammarstedt et al., 2018; Chouchani and Kajimura, 2019; Guzman-Ruiz et al., 2020). Increased deposition of ECM proteins such as collagens (e.g., collagen I, III, IV, VI), fibronectin, and elastin in adipose tissues is associated with infiltration of proinflammatory immune cells, which leads to adipose tissue disorganization and dysfunction in obese conditions (Chouchani and Kajimura, 2019). It is reported that increased adipose tissue fibrosis in the subcutaneous adipose tissues contributes to insulin resistance and metabolic disorder (Sun et al., 2013). ECM stiffness also controls angiogenesis (Mammoto et al., 2009). Since Twist1 is a mechanosensitive gene and senses ECM stiffness (Fattet et al., 2020) and contributes to mechanosensitive pathology [e.g., pulmonary fibrosis (Mammoto et al., 2016), pulmonary hypertension (Mammoto et al., 2018, 2020), cancer (Fattet et al., 2020), atherosclerosis (Mahmoud et al., 2016, 2017)], Twist1 may sense changes in the ECM microenvironment in the obese adipose tissues and control angiogenesis and adipogenesis.

Obesity-mediated inflammation and lipotoxicity through ectopic lipid deposition contribute to vascular remodeling in ectopic organs (e.g., liver, muscle, pancreas, heart) as well as in tumor tissues, promoting insulin resistance (Kim et al., 2007; Savage, 2009; Virtue and Vidal-Puig, 2010), cardiovascular diseases and tumor progression (Dong et al., 2017). Regarding angiogenesis in obese subcutaneous adipose tissues, consistent with our results, it is demonstrated that angiogenesis is impaired and endothelial function is disrupted in obese subcutaneous adipose tissues, which results in ectopic lipid accumulation and obesity-associated diseases (Hosogai et al., 2007; Halberg et al., 2009; Pasarica et al., 2009; Cao, 2010; Christiaens and Lijnen, 2010; Corvera and Gealekman, 2014; Shimizu et al., 2014; Fuster et al., 2016; Xiao et al., 2016; Crewe et al., 2017; Hammarstedt et al., 2018; Chouchani and Kajimura, 2019). Under obese conditions, due to its proinflammatory state, endothelial signaling and their functionality are impaired (Hosogai et al., 2007; Halberg et al., 2009; Pasarica et al., 2009; Cao, 2010; Christiaens and Lijnen, 2010; Corvera and Gealekman, 2014; Shimizu et al., 2014; Fuster et al., 2016; Xiao et al., 2016; Crewe et al., 2017; Hammarstedt et al., 2018; Chouchani and Kajimura, 2019). Multiple groups have reported that stimulation of angiogenesis in adipose tissue of obese rodents by increasing angiogenic gene expression (e.g., VEGFA, VEGFB, FLT1, FOXO, Angiopoietin2) not only improves local adipose tissue function, but also counteracts systemic metabolic disorders (Sun et al., 2012; Sung et al., 2013; Robciuc et al., 2016; An et al., 2017; Rudnicki et al., 2018; Seki et al., 2018). Thus, the response of ECs to obesity and associated inflammatory and angiogenic signaling may be different among organs and obesity/disease stages due to heterogeneity of ECs and differences in the microenvironment (Mammoto et al., 2009; Mammoto and Mammoto, 2019; Ren et al., 2019). Further time course analysis and investigation of inflammatory gene expression and immune cell infiltration in subcutaneous adipose tissue may elucidate the mechanism.

In this report, we isolated ECs from human subcutaneous adipose tissues of lean vs. obese individuals, which includes a variety of other conditions. Since aging affects angiogenesis (Mammoto et al., 2019a; Hendee et al., 2021), we only used ECs from tissues of young patients (<55 years old). Recent lineage tracing mouse study revealed that subcutaneous adipose tissue expansion pattern is sex dependent; while subcutaneous adipose tissue expansion is through hypertrophy in male mice, increase in adipose tissue is through the combination of hypertrophy and hyperplasia in female mice (Jeffery et al., 2016; Chouchani and Kajimura, 2019). Thus, the pattern and signaling mechanism of adipogenesis and angiogenesis in adipose tissue may be different due to sex, the onset of obesity, and cardiovascular conditions. However, due to tissue availability, most of the human samples in this study are from females. Investigation using a different cohort and a larger sample size will further elucidate the mechanism.

In summary, we have found that vascular formation is inhibited in ECs isolated from obese human subcutaneous adipose tissues through TWIST1-SLIT2 signaling. Modulation of endothelial TWIST1-SLIT2 signaling may be an effective strategy for treating obesity and associated metabolic complications.

## Data Availability Statement

Publicly available datasets were analyzed in this study. This data can be found here: NCBI GEO, GSE55200.

## Ethics Statement

The studies involving human participants were reviewed and approved by Institutional Review Board of MCW and Froedtert Hospital. The patients/participants provided their written informed consent to participate in this study. The animal study was reviewed and approved by Animal Care and Use Committee of MCW.

## Author Contributions

TM and AM conceived and designed the experiments. TH, KH, KM, PK, TM, and AM performed the experiments. TH, KH, TM, and AM analyzed the data, contributed reagents, materials, and analysis tools, and wrote the manuscript. All the authors contributed to the article and approved the submitted version.

## Conflict of Interest

The authors declare that the research was conducted in the absence of any commercial or financial relationships that could be construed as a potential conflict of interest.

## Publisher’s Note

All claims expressed in this article are solely those of the authors and do not necessarily represent those of their affiliated organizations, or those of the publisher, the editors and the reviewers. Any product that may be evaluated in this article, or claim that may be made by its manufacturer, is not guaranteed or endorsed by the publisher.

## Supplementary Material

Supplementary Figure 1 TWIST1 controls EC survival in human adipose ECs. **(A)** Representative images showing TWIST1 expression and DAPI in lean (BMI < 30) human adipose ECs treated with TWIST1 siRNA (*left*). As a control, human adipose ECs were treated with siRNA with irrelevant sequences. Scale bar, 10 μm. Graph showing integrated density of TWIST1 (*right*, *n* = 5, mean ± SEM, ^∗^*p* < 0.05). **(B)** Representative images showing trypan blue staining of lean (BMI < 30) human adipose ECs treated with TWIST1 siRNA. Scale bar, 25 μm. Graph showing trypan blue excluding lean human adipose ECs treated with TWIST1 siRNA (*n* = 5, mean ± SEM, ^∗^*p* < 0.05). As a control, human adipose ECs were treated with siRNA with irrelevant sequences.

Supplementary Figure 2 TWIST1-SLIT2 signaling mediates obesity-dependent inhibition of vascular formation in the gel subcutaneously implanted on mice. **(A)** Fibrin gel subcutaneously implanted on the back of a mouse. Scale bar, 1 mm. Arrow indicates the implanted gel. **(B)** 3D reconstructed IF images showing GFP-labeled and VEGFR2-stained vascular formation and DAPI in the fibrin gel supplemented with GFP-labeled lean (BMI < 30, clone #15) vs. obese (BMI > 30, clone #7) human adipose ECs and subcutaneously implanted on NSG mice for 7 days (*top*). 3D reconstructed IF images showing GFP-labeled and VEGFR2-stained vascular formation and DAPI in the fibrin gel supplemented with GFP-labeled lean (BMI < 30, clone #15) human adipose ECs treated with SLIT2 shRNA and subcutaneously implanted on NSG mice for 7 days (*bottom*). Scale bar, 50 μm. **(C)** 3D reconstructed IF micrographs showing GFP-labeled and VEGFR2-stained vascular formation and DAPI in the fibrin gel supplemented with GFP-labeled obese human adipose ECs (clone #7) treated with SLIT2 protein, lentivirus overexpressing TWIST1 or in combination with SLIT2 shRNA and subcutaneously implanted on NSG mice for 7 days. As a control, obese human adipose ECs were treated with control virus (vector alone), control shRNA, or control vehicle. Scale bar, 50 μm.
