# Supplementary figures and images for: Obesity Inhibits Angiogenesis Through TWIST1-SLIT2 Signaling
Source: Front Cell Dev Biol. 2021 Sep 29;9:693410. doi: 10.3389/fcell.2021.693410 (PMC8511494; doi:10.3389/fcell.2021.693410)

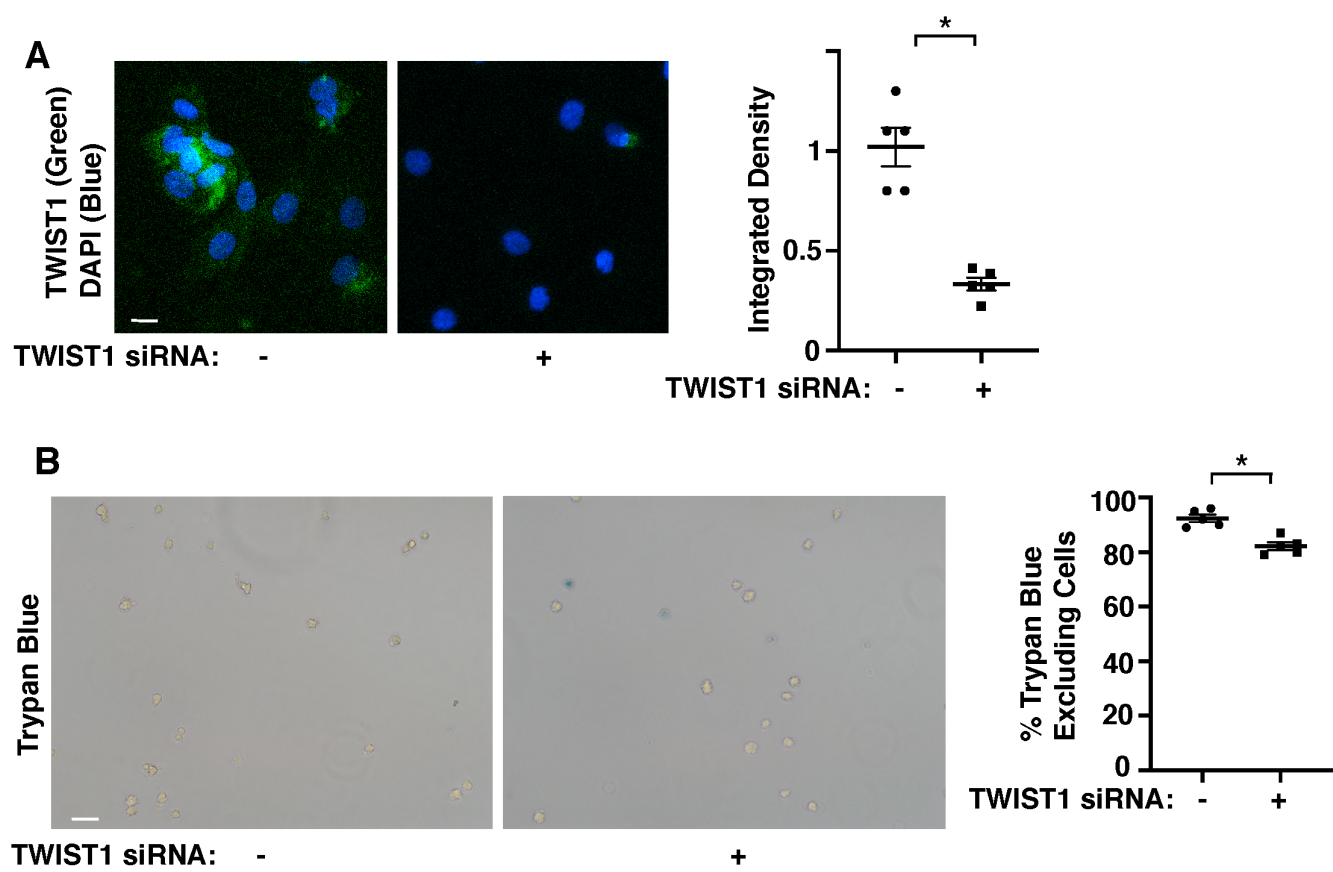

Supplementary Fig. 1

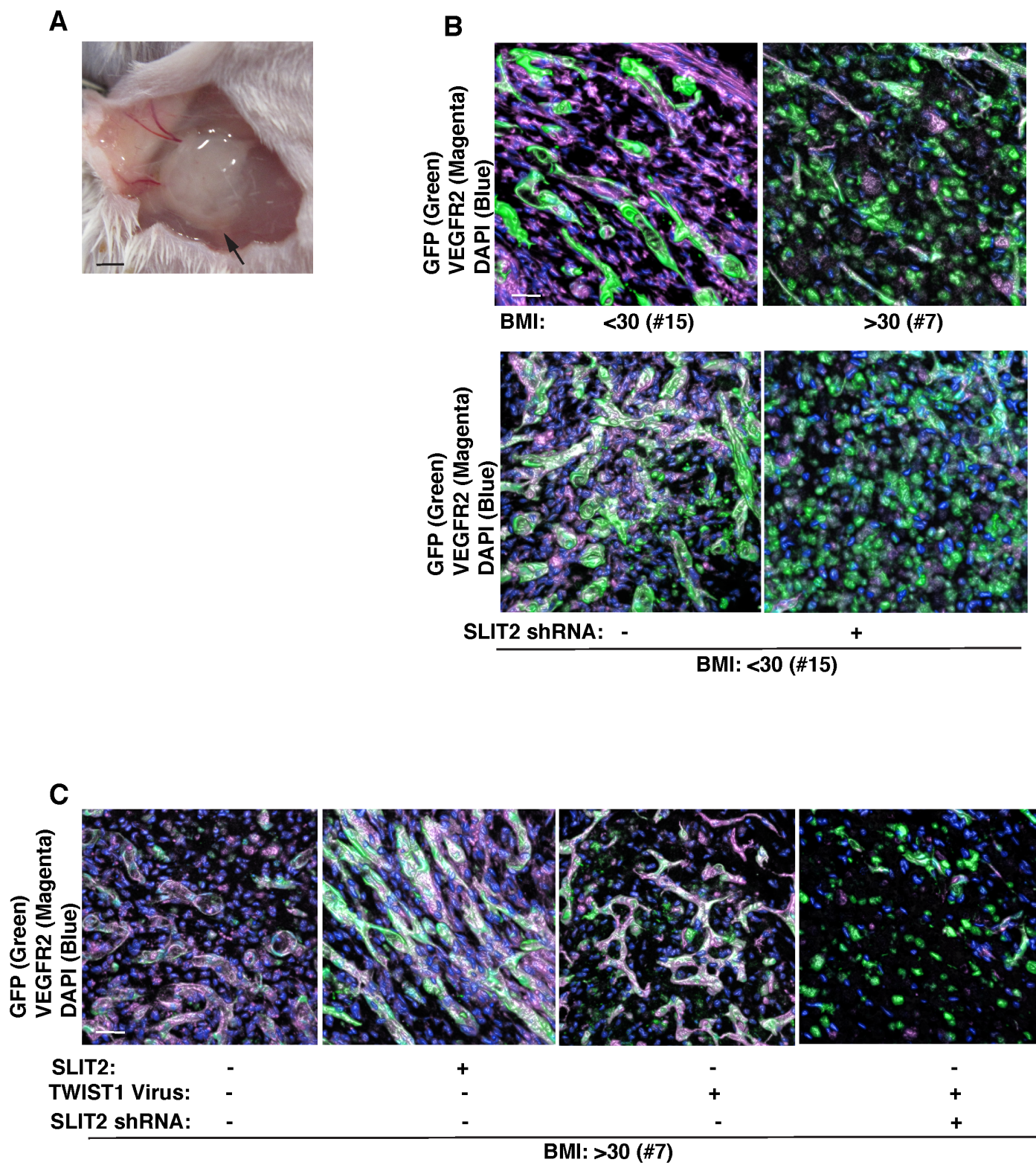

Supplementary Fig. 2

Supplement: Supplementary Figure 1 — TWIST1 controls EC survival in human adipose ECs. (A) Representative images showing TWIST1 expression and DAPI in lean (BMI < 30) human adipose ECs treated with TWIST1 siRNA (left). As a control, human adipose ECs were treated with siRNA with irrelevant sequences. Scale bar, 10 μm. Graph showing integrated density of TWIST1 (right, n = 5, mean ± SEM, ∗p < 0.05). (B) Representative images showing trypan blue staining of lean (BMI < 30) human adipose ECs treated with TWIST1 siRNA. Scale bar, 25 μm. Graph showing trypan blue excluding lean human adipose ECs treated with TWIST1 siRNA (n = 5, mean ± SEM, ∗p < 0.05). As a control, human adipose ECs were treated with siRNA with irrelevant sequences. [file Data_Sheet_1.PDF]
